# Mechanical Metamaterial‐Based Structure with Magnetically Controlled Nonreversibility and Nonreciprocity for Programmable Locomotion

**DOI:** 10.1002/advs.202503088

**Published:** 2025-06-19

**Authors:** Krzysztof K. Dudek, Olly Duncan, Julio A. Iglesias Martínez, Muamer Kadic

**Affiliations:** ^1^ Institute of Physics University of Zielona Gora ul. Szafrana 4a Zielona Gora 65‐069 Poland; ^2^ Department of Engineering Manchester Metropolitan University Manchester M1 5GD UK; ^3^ Institut Jean Lamour University Lorraine CNRS UMR 7198 Nancy Cedex 54011 France; ^4^ Université Marie et Louis Pasteur Institute FEMTO‐ST Besançon 25000 France

**Keywords:** mechanical metamaterials, magneto‐mechanical, nonreciprocal

## Abstract

Programmable mechanical metamaterials hold the key to critical innovations in materials science research, by harnessing relatively unexplored nonlinearities that change effective responses. Effective properties of a metamaterial strongly depend on the reversibility of the deformation process. While most elastic materials show reciprocity and reversibility, the possibility of concurrently observing nonreciprocity, defined as a deformation that is not mirrored when a body is loaded equally from opposite sides, as well as a nonreversible deformation process, opens doors to addressing complex mechanical problems that are crucial from the perspective of soft body dynamics. In this work, a magneto‐mechanical metamaterial‐based structure is proposed that simultaneously exhibits both of these phenomena by utilizing elastic and magnetically induced nonlinearities. It is shown that such a system can undergo a transition in its static mechanical properties, such as Poisson's ratio and stiffness, leading to stark changes in energy absorption. It is also demonstrated that, thanks to the asymmetric distribution of magnetic inclusions, the entire structure can exhibit an efficient locomotion mechanism suitable for applications in robotics.

## Graphical abstract

A magneto‐mechanical metamaterial‐based structure that simultaneously exhibits programmable nonreversibility and nonreciprocity. These cause substantial transitions in static mechanical properties, such as Poisson's ratio and stiffness, leading to stark changes in energy absorption. Using an asymmetric distribution of magnetic inclusions, the entire structure can exhibit an efficient locomotion mechanism suitable for applications in robotics.

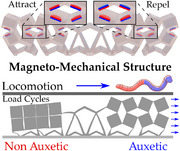

## 1 Introduction

Reciprocity and reversibility govern classical elasticity.^[^ ^1^, ^2^, ^3^, ^4^, ^5^, ^6^ ^]^ The Maxwell‐Betti theorem^[^ ^7^, ^8^, ^9^ ^]^ states that the displacements of two points should be mirrored when each point is subject to the same load,^[^ ^1^ ^]^ and is ubiquitously applied in mechanics.^[^ ^10^, ^11^, ^12^ ^]^ The defining characteristic of an elastic material is that the force and deformation should be reversible when loaded or unloaded.^[^ ^10^, ^11^ ^]^ These relationships govern the usual degrees of freedom that are considered in material design and selection, but can be broken by designing substantial structural reconfiguration into material structures. One of the prime classes of systems that can particularly benefit from breaking reciprocity and reversibility are mechanical metamaterials.

Mechanical metamaterials^[^ ^12^, ^13^, ^14^, ^15^, ^16^, ^17^, ^18^, ^19^, ^20^, ^21^ ^]^ are rationally designed structures that exhibit unusual mechanical properties. Mechanical metamaterials have been intensely studied from the perspective of their exotic properties, such as auxetic behavior,^[^ ^17^, ^22^, ^23^, ^24^, ^25^, ^26^ ^]^ negative stiffness,^[^ ^27^, ^28^ ^]^ superior functionality related to energy absorption efficiency,^[^ ^29^ ^]^ and controllable wave attenuation and dispersion.^[^ ^30^, ^31^, ^32^, ^33^, ^34^ ^]^ While mechanical metamaterials have been successfully implemented in applications ranging from protective equipment to biomedical devices,^[^ ^35^ ^]^ most cannot substantially change their properties without being redesigned and reproduced. Designing active, programmable mechanical metamaterials allows wider applications in growing markets, such as robotics or flexible electronics. These active mechanical metamaterials can be controlled via various stimuli such as magnetic fields,^[^ ^36^, ^37^, ^38^, ^39^, ^40^ ^]^ temperature,^[^ ^41^, ^42^, ^43^, ^44^ ^]^ or light.^[^ ^45^, ^46^, ^47^ ^]^ The concept of using an internal actuation mechanism paves a new path toward achieving new programmable responses, such as nonreciprocal, and non‐reversible deformations, via. control of these internal interactions. While active and passive mechanical metamaterials have recently been shown to be capable of breaking reciprocity,^[^ ^1^, ^3^, ^4^, ^5^, ^6^, ^48^, ^49^, ^50^, ^51^, ^52^ ^]^ and viscoelastic, or bistable metamaterials can break reversibility,^[^ ^4^, ^16^, ^53^ ^]^ actively controlled metamaterials may open the door to the idea of observing and controlling nonreciprocity and nonreversibility simultaneously.

Nonreciprocal mechanical metamaterials harness material nonlinearities to change effective response when a load is applied from opposing points. For example, a “fishbone” structure will have different stiffness when displacement of its central spine causes its oblique ribs to open or close.^[^ ^1^, ^5^, ^6^, ^48^, ^49^, ^51^, ^52^ ^]^ Nonreciprocity has also been achieved under distributed loads, using active programmable mechanical metamaterials (whereby the nonreciprocal condition is controlled by an external energy source^[^ ^2^, ^3^ ^]^), or in passive mechanical metamaterials with bi‐stability.^[^ ^4^ ^]^ Given these advancements, the question that arises is whether structural nonlinearities can be utilized concurrently with the nonreversibility of the deformation process induced, for example, by interactions between adjacent structural units. This could help to design active metamaterials capable of not only changing their static mechanical properties such as Poisson's ratio, which could be attributed to a change in the reconfiguration pattern induced by the lack of reversibility, but also exhibiting additional phenomena, such as programming internal and external structural bias, leading to locomotion.

Efficient locomotion and shielding from potentially damaging loads are core requirements in soft robotics, biomedical equipment, and protective equipment.^[^ ^54^, ^55^, ^56^ ^]^ These relate directly to reversibility and reciprocity; depending on the location of a contact, a different response may be required. Similarly, controlling reversibility allows a transition between a low‐loss impact and a low‐impulse one; respectively associated with locomotive efficiency,^[^ ^35^ ^]^ or improved protective capacity.^[^ ^54^ ^]^ Developments in such capabilities cause market disruption. For example, “super‐shoes” with a claimed 4% increase in running efficiency have revolutionized athletics.^[^ ^35^, ^57^ ^]^ As such, the capability to control and direct force/displacement vectors has great potential.^[^ ^2^ ^]^

In this work, we propose a novel magneto‐mechanical metamaterial designed to harness both elastic and magnetically induced nonlinearities, causing controllable nonreversibility and nonreciprocity. This concept provides new design capabilities that allow stark changes in static and dynamic mechanical properties, such as stiffness (from positive to negative) and Poisson's ratio (from positive to negative). The ability to impart a structural bias using magnetic inclusions, and to amplify its effect using controllable nonreversibility and nonreciprocity, enables controllable locomotion during cyclic loading.

## 2 Concept

### 2.1 Reversible and Non‐Reversible Deformation

**Figure 1:**
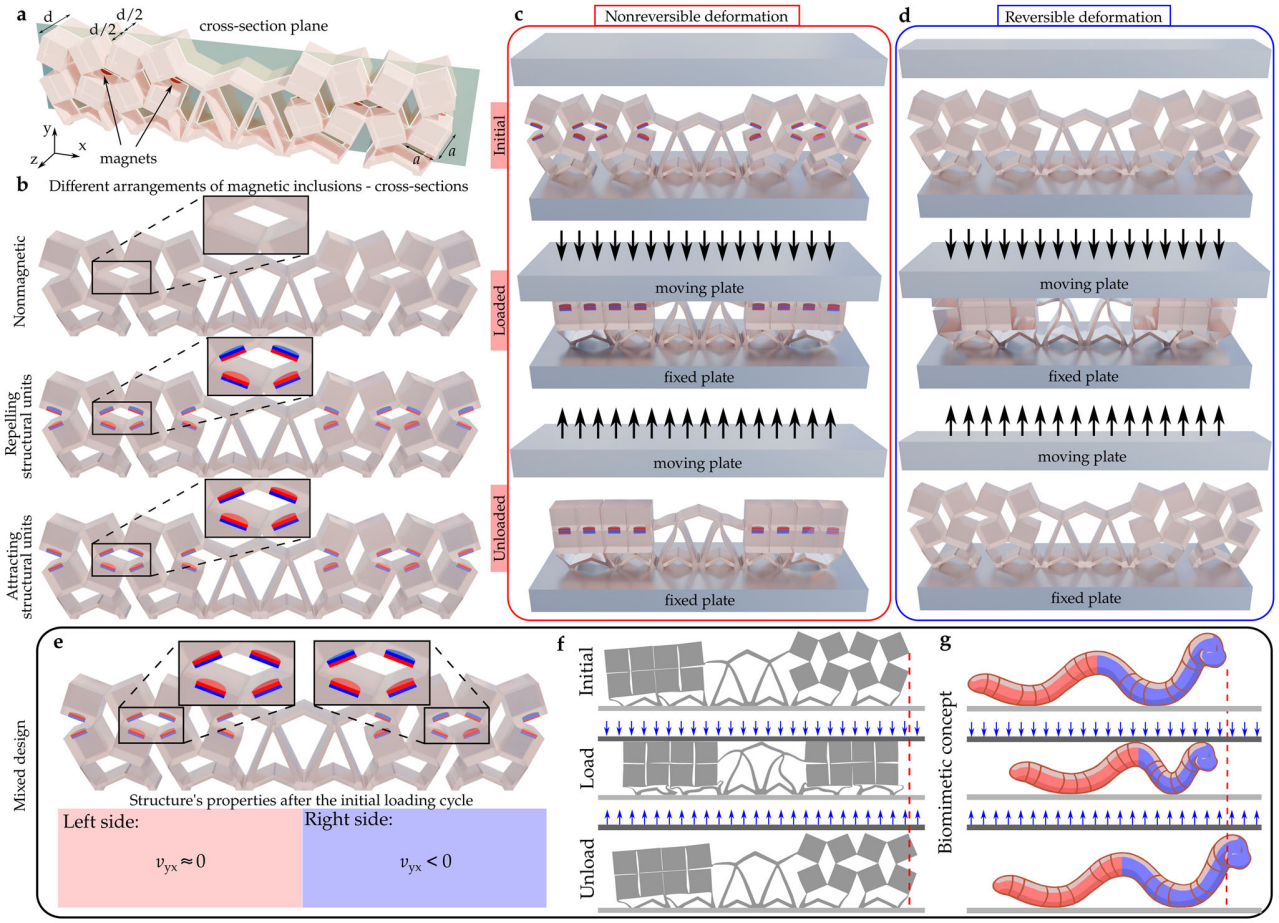
Design of the magneto‐mechanical structure. a) Considered model. The cross‐section plane is used to demonstrate the orientations of magnets within the structure. b) The three types of structure considered in this work, with all of them having a symmetric design resulting in the left and right‐hand sides of the system being identical. These designs vary in terms of their composition: i) the nonmagnetic structure, ii) the system incorporating magnets arranged in a manner resulting in their effective repulsion, iii) the structure with attracting magnets. c) Graphical representation of the nonreversible deformation process corresponding to the model with mutually attracting adjacent magnetic inclusions. d) Reversible deformation of the non‐magnetic structure. e) System consisting of magnets arranged in the attracting and repelling configuration on the left‐ and right‐hand sides, respectively. f) Conceptual visualization of the deformation process of the structure with a left‐right biased design that results in the translation of the entire system. g) A biomimetic representation of the concept describing a translation of the metamaterial‐based structure having a left‐right biased design, which we later show also leads to a nonreciprocal response.

The concept of our model that can be programmed to switch between a reversible and non‐reversible deformation was achieved using an internal actuation mechanism, based on magnetic interactions. The novel magneto‐mechanical metamaterial‐based structure combined an elastic lattice and magnetic inclusions; cylindrical neodymium magnets (see **Figure** **1**a). The structure consists of both solid square‐like rigid blocks (having a side length of *a* = 2 cm), resembling the well‐known auxetic rotating squares system,^[^ ^24^ ^]^ with connecting walls. Around the central part of the system, fragments of these walls resemble a negative Poisson's ratio arrowhead metamaterial,^[^ ^25^ ^]^ although the geometry has been modified to exhibit positive Poisson's ratio. This means that both the left and the right‐hand side of this structure are expected to have a negative Poisson's ratio, while the middle of the system is expected to have a positive Poisson's ratio. Due to the complexity of the geometry, specific dimensions are provided in the Supporting Information (S2). Here, it should be noted that in our analysis, we treat the considered model as a finite structure, consisting of the two types of lattice based metamaterials. However, although we do not pursue such analysis in our study, it could also be considered as half of a unit cell of a larger mechanical metamaterial.

The arrangement of magnets within the system is shown in the auxiliary *xy* plane that passes through the center of the metamaterial‐based structure (Figure 1a). As shown in Figure 1b, we consider three arrangements of magnetic inclusions. Namely, the nonmagnetic case, as well as structures with repelling and attracting magnets. The main motivation behind these arrangements corresponds to differences in the actuation mechanisms between the resulting structures. Namely, they can either aid the rotations of structural blocks (attracting magnets), resist them (repelling magnets), or not affect them at all (no magnets) during loading (or vice versa while unloading). This internal actuation is used to control both the deformation patterns and the static mechanical properties.

As shown in Figure 1c,d, these differences in the reconfiguration become apparent when comparing the response of the nonmagnetic model and the structure with attracting magnets. Both structures are compressed along the *y*‐axis (N.B., the same type of compression is applied to induce the reconfiguration of all structures considered in this work ‐ see Experimental Section). According to Figure 1c,d, when being unloaded, it becomes evident that the deformation of the system with attracting magnets is highly nonreversible, when the magnets become close enough so that their relative magnetic interactions overcome elastic forces related to the nonmagnetic structure. Conversely, the deformation of the nonmagnetic structure is fully reversible.

### 2.2 Mixed Composition ‐ Locomotion Mechanism

As described above, to analyze the effect of the reciprocal deformation and its influence on static mechanical properties of the metamaterial‐based model, we consider structures where the arrangement of magnetic inclusions is symmetric (i.e., the same on the left and right of the structure). To study the potential of a locomotive model, that is appealing from the perspective of applications in robotics, we impart a bias in the structure by reversing this left‐right symmetry. As shown in Figure 1e, we arranged the left and right‐hand side magnets to be, respectively, attracting or repelling. This results in a strongly biased deformation mechanism. Figure 1f clearly shows that, after the initial loading cycle, blocks with attracting magnets are confined in a locked configuration, meaning that the left and right‐hand sides of the system have substantially different Poisson's ratios. In Figure 1g, we show (conceptually) through the use of a schematic biomimetic model how the deformation of the structure with this inbuilt bias could exhibit locomotion, related to appropriately timed transverse contraction or extension of the left or right side of the structure. Specific details related to the origin and mechanism responsible for the locomotion mechanism are provided in the Results and Discussion section (see Videos S2 and S3, Supporting Information), with further details provided in the Supporting Information (Section 5).

## 3 Results and Discussion

### 3.1 Control Over Mechanical Properties of the Structure

**Figure 2:**
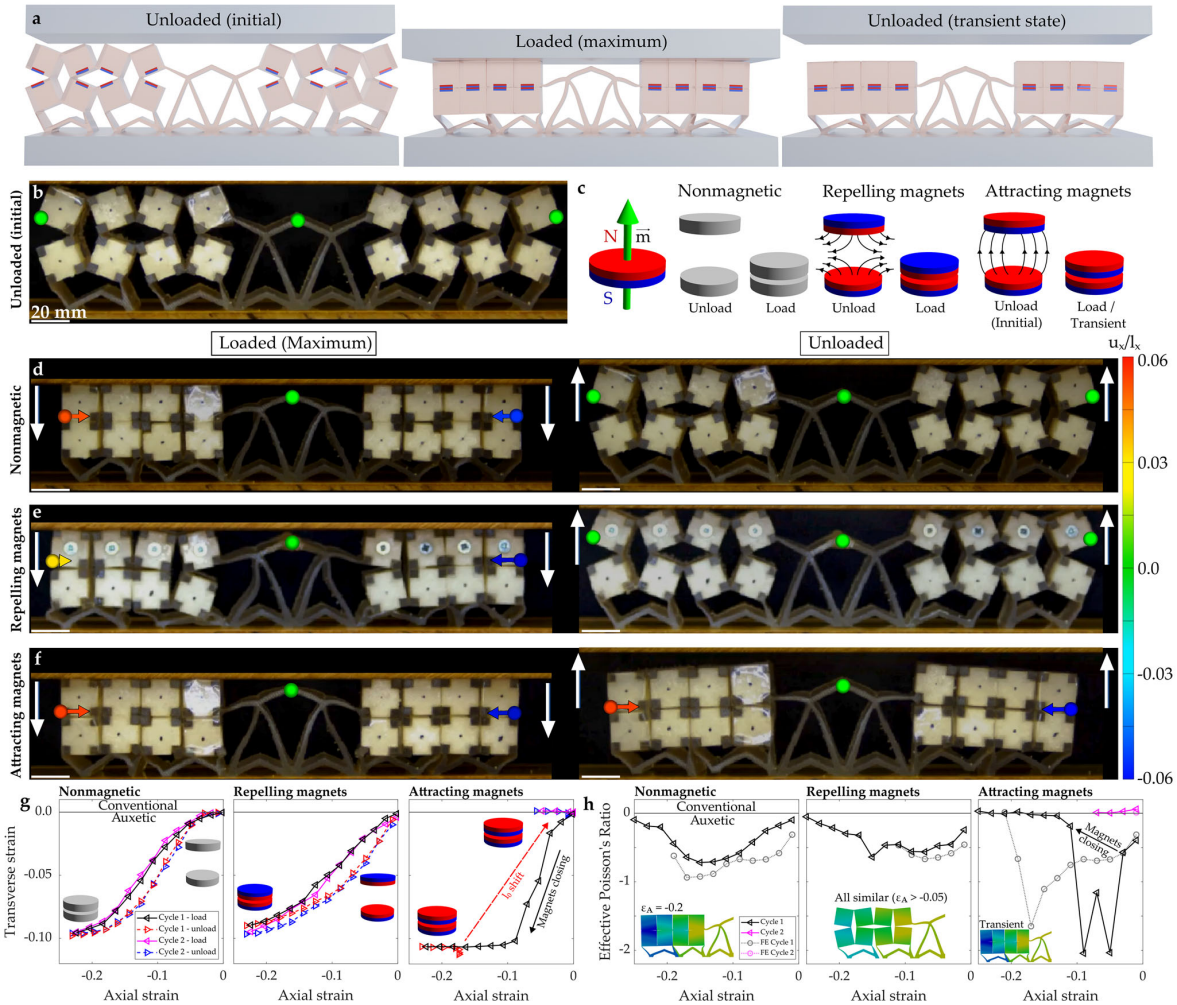
Results corresponding to the quasistatic deformation of the three types of structures analyzed in this work from the perspective of their static mechanical properties. a) Graphical representation of the loading/unloading cycle for the structure with attracting magnets. b) Initial configuration of the system which, in terms of its geometry, is the same irrespective of the types of arrangements of magnets. c) Magnet configurations. d) to f) Tracking and vector plots of the final configuration of experimental samples subject to transverse deformation when loaded (LHS: left‐hand side) and unloaded (RHS: right‐hand side). The three tracked samples correspond to different arrangements of magnetic inclusions: d) no magnets, e) magnets in repel, f) magnets attracting (see Video S1, Supporting Information). The legend on the RHS from panels d,e), applies to vectors on panels b,d–f), as well as false color plots in h), and indicates the relative transverse translation of the specific parts of the structure compared to their initial positions. Here, *u*_*x*_ is related to the translation of a given point along the *x*‐axis (relative to its initial position) and *l*_*x*_ is the initial horizontal dimension of the entire structure. g) Transverse versus axial true strain, and h) incremental Poisson's ratio vs axial strain. Legends shown on the first graphs of panels g) and h) apply to all graphs on each of these panels.

The arrangement of magnetic inclusions (as shown in **Figure** **2**a–c) considerably influences the deformation process (Figure 2c,d–f). When the magnets are excluded or repelling, the metamaterial‐based structure returns to the initial configuration at the end of each loading cycle. The attracting magnets prevent this recovery, by holding the square structural blocks in direct contact. This prevention of reversibility in the deformation process has consequences for the structure's Poisson's ratio. One can note that the horizontal dimensions of each configuration visibly shrink during compression along the *y*‐axis (Figure 2d–f, left‐hand side) which is indicative of auxetic behavior. As the samples are unloaded, however, stark differences can be seen. For the nonmagnetic configuration and the system composed of repelling magnets (Figure 2d,e, right‐hand side), one can observe gradual horizontal expansion, again indicative of a negative Poisson's ratio. Conversely, as the sample with attracting magnets is unloaded, its horizontal dimension becomes almost unchanged (Figure 2f right‐hand side), indicative of a Poisson's ratio of approximately zero.

According to Figure 2g, both the nonmagnetic structure and the sample with repelling magnets exhibited auxetic behavior throughout loading and unloading, reaching values of approximately –1 (Figure 2g,h). As the polymer used was highly viscoelastic, as shown in the Supporting Information (Figure S2), some hysteresis was present in these quasi‐static tests. The difference between load/unload for the nonmagnetic case forms a baseline for comparison to other cases. The sample with attracting magnets, however, was strongly auxetic at the beginning of the first loading cycle (with Poisson's ratio as low as –2, Figure 2h). As the loading process progressed, incremental Poisson's ratio (ν_*yx*_) quickly increased to values marginally higher than zero when the magnets came into contact. Subsequently, during the unloading process, the Poisson's ratio remained close to zero (i.e., negligible transverse strain, Figure 2g) ‐ a change between the load/unload stage that substantially exceeds the hysteretic effect seen in the nonmagnetic case. This behavior changes during the second deformation cycle, when Poisson's ratio remains marginally positive, as the consistent strong attractive forces between the magnets prevent the “rotating squares” from reopening. Interestingly, this means that after the initial nonreversible deformation cycle, this process becomes reversible, although the range of the structure's vertical motion is strongly diminished.

**Figure 3:**
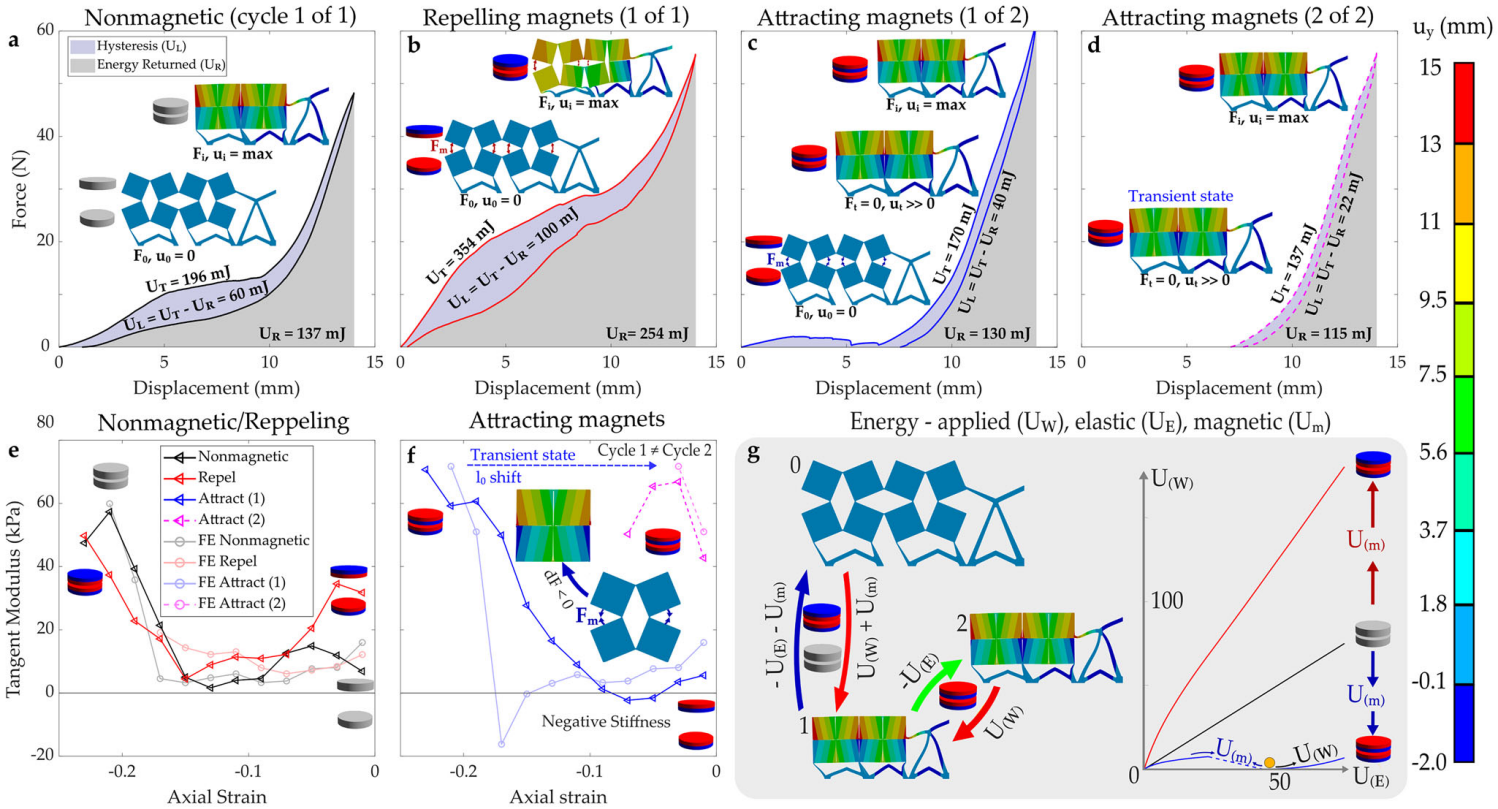
Energy stored by the structures with the symmetric design (LHS and RHS of the system are the same), portrayed in Figure 1b, subjected to the quasistatic deformation process. a–d) Graphs of the reaction force vs. displacement recorded during experiments in the case of samples having a different composition: a) no magnets, b) repelling magnets, c) attracting magnets (cycle 1 out of 2), and d) attracting magnets (cycle 2 out of 2 ‐ two cycles are portrayed solely for the system with attracting magnets as for other structures there are no major differences between the consecutive cycles). Here, the shaded regions represent the energy absorbed during the loading process (whole shaded region, *U*_*T*_), the energy released as the samples were unloaded (gray region, *U*_*R*_), and the hysteresis (purple region, *U*_*L*_). Legend on panel a) applies to all graphs from panels a–d). e) and f) Tangent modulus vs. axial strain for the system with e) no magnets and repelling magnets, and f) attracting magnets. g) Simulation outputs showing undeformed (labeled ”0”), deformed (labeled ”1”), and transient states (labeled ”2”), controlled by magnet configurations. The arrows show the amount of applied deformation/resultant recovery required to switch between each state. The RHS of this panel shows a plot of applied vs. stored elastic and magnetic energy, and the energy‐well contributing to the transient state when magnets are attracting (cropped to focus on the transition region). The symbols *F* and *U* denote force or energy, respectively. The subscripts subscripts *W*, *E*, and *m* denote respective externally applied, elastic, or magnetic effects. The color bar on the right‐hand side of the figure applies to the false color plots in all panels. Here, *u*_*y*_ represents the vertical displacement.

The nonmagnetic system shows nonlinear force versus displacement, with a relative force plateau followed by substantial stiffening (when the squares come into self‐contact **Figure** **3**a), and a moderate level of hysteresis due to the polymer viscoelasticity. The system with repelling magnets is stiffer than the nonmagnetic one, with greater hysteresis (Figure 3b), while the system with attracting magnets is substantially less stiff, with a negative stiffness (snap‐through) region in the first loading cycle (Figure 3c). When the attracting magnets remain connected, a transient state is realized, whereby the rotating squares are locked together and the response is caused by deformation of the other structural components. This results in a slight reduction in energy absorption and a stark reduction in subsequent hysteresis (Figure 3d). When the magnets are attracting, the compression plates remain in contact with the structure for a portion of the first unloading phase (between 7 and 14 mm), and over the same portion of the loading and unloading phase for subsequent cycles (as seen in Video S1, Supporting Information). The resulting force is caused by the strain energy being absorbed by or released from the structure surrounding the rotating squares. Later during the unloading phase, separation occurs between the compression plates and the sample, as the structure surrounding the squares mostly recovers its initial shape while the squares are still constrained, noting that the shape of the elements surrounding the squares cannot be completely recovered due to the connection with the locked squares. This scenario can be observed for displacements smaller than 7 mm, when the resultant reaction force is zero (Figure 3c,d).

The repelling magnets (at first) increase stiffness, which then reduces (for axial strains < –0.15, Figure 3e), when the squares are held apart by the magnets (Figure 3b (inset), or Figure 2e (RHS)). This lack of stability in large deformations increases hysteresis, as repelling magnets become misaligned (Figures 2e and 3b), reducing the contribution of the magnetic force to recovery.^[^ ^58^, ^59^ ^]^ This change in load/unload is another example of a nonreversible deformation, that is beyond any expected viscoelastic effects (i.e., double the hysteresis of the nonmagnetic structure in Figure 3a). The attracting magnets have the opposite effect, reducing the stiffness (to negative values) at smaller deformations, then increasing stiffness during the self‐contact region, and subsequent loading cycles (when the magnets remain connected, Figure 3f). This causes a large nonreversible deformation on the first cycle, as previously shown in Figure 2.

**Figure 4:**
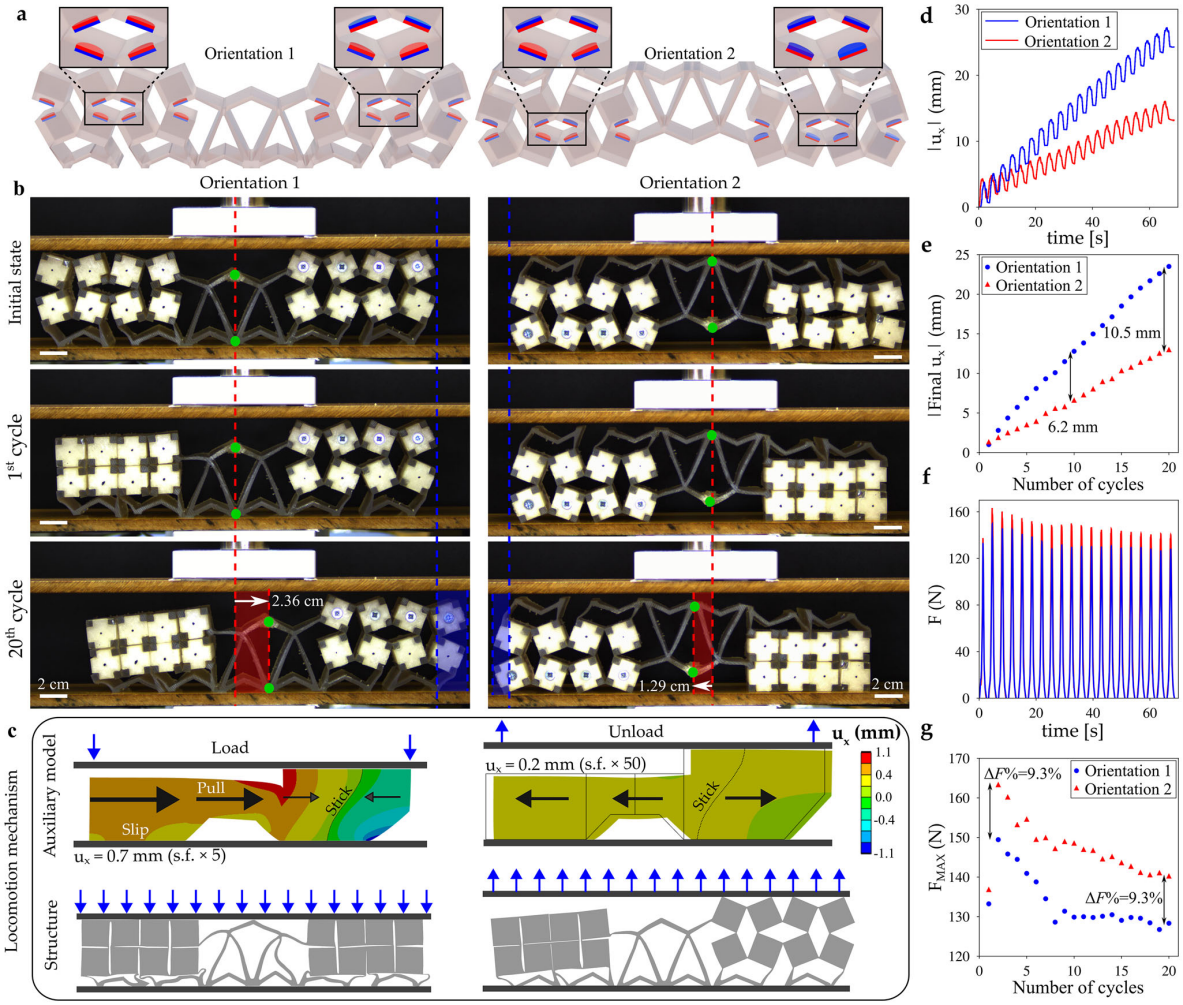
a) The two orientations of the system chosen to investigate its reciprocity. In both cases, the structure has a mixed composition in terms of magnetic inclusions, and dynamic cyclic compression tests were applied. b) Configurations assumed by the metamaterial‐based model in both orientations, before cyclic compression, then after the 1^*st*^ and 20^*th*^ respective compression cycles. Translation of the center of the system caused by the cyclic deformation procedure is indicated using a dashed red line and semitransparent red region, while that of the side containing repelling magnets is blue (see Videos S2 and 3, Supporting Information). c) Diagrams showing the Finite Element Model results for the auxiliary model, which is a simplified version of the considered structure. This model explains the locomotion mechanism of the system observed in the experiments. For better visibility, graphical representation of the structure's translation is emphasized by applying a scale factor (s.) of 50, so that it can be clearly observed compared to the initial outline. d) Experimental results corresponding to the translation of the middle middle part of the system plotted against time for both of its orientations. e) Translation of the structure at the end of every loading cycle. f) Reaction force measured in the experiment plotted against time. g) Maximum reaction force recorded during every loading cycle.

The stark change between loading and unloading when the attracting magnets first connect is a nonreversible deformation. It is this response, which temporarily changes the shape of the structure, as well as sensitivity to the point of application of dynamic deformations, that are later targeted to the break reciprocity condition in the Maxwell–Betti theorem (in **Figure** **4**). The transient state, caused by the attracting magnets, is effectively a local minimum (or “well”) between elastic energy and applied energy (Figure 3g ‐ RHS), and is expected to be stable despite any small changes in initial conditions. Here, the energy stored in the magnets exceeds that of the elastic energy stored in the hinges of the rotating squares. The stiffening caused by the repelling magnets can similarly be explained by the contribution of the magnetic field to the applied energy.

### 3.2 Locomotion

To analyze the potential of the proposed structure to exhibit locomotion, we implement a biased design. This bias is created by setting left‐hand side magnets to be attracting, and right‐hand side magnets to repel. We then vary initial conditions, to affect the orientation (and interaction) of repelling magnets, by using two different (flipped) spatial orientations (Orientation 1 and 2, Figure 4a). After twenty consecutive loading/unloading cycles, which were dynamic (as required to overcome frictional dependencies ‐ see Section S5, Supporting Information), both orientations do indeed shift relative to their initial positions (Figure 4b). The extent and direction of the translation varied for both orientations. Namely, the middle part of the structure in Orientation 1 moved to the right by 2.4 cm, while the system with the opposite orientation moved 1.3 cm to the left (i.e., both moving toward the side with the repelling magnets). After the first loading/unloading cycle, the attracting magnets locked the square structural blocks on one side of the system together, throughout the remaining deformation cycles.

The observed locomotion mechanism was reliable, and the extent of translation between the consecutive loading/unloading cycles remained approximately the same (Figure 4d–e). The structure strongly translated in the direction matching that of the overall motion at maximum compression, and then partially shifted back during the unloading process (Figure 4d). The global translation was caused by the translation during the loading stage exceeding the returning translation during the unloading stage. More specifically, in the case of Orientation 1, after every deformation cycle, the center of the system shifted by 3.92 ± 0.15 mm (mean ± standard deviation) to the right when loaded, then returned by −2.71 ± 0.15 mm when unloaded. For orientation 2, cyclic translations were −3.42 ± 0.10 mm and −2.71 ± 0.08 mm respectively. As such, after all cycles, the overall translations for orientations 1 and 2 were 2.4 cm and –13 cm, respectively.

As with global locomotion, the change in the orientation also affects the reaction force (Figure 4f–g). Namely, according to Figure 4g, the maximum reaction force is consistently larger in Orientation 2. The difference between the maximum reaction forces is approximately 9 % for all deformation cycles, confirming that the Maxwell‐Betti reciprocity theorem is broken. As discussed in the Supporting Information (Section S5), the change in response resulting from the change in the location of the applied deformation is related to the sensitivity of the left‐side of the biased structure to friction and dynamics. When the deformation signal is initiated from above (Orientation 1), the dynamic effects align with forces and moments acting on the structure, as shown in the Supporting Information (Section S4). When initiated from below, these dynamic effects are reversed. This results in a change in locomotion (Figure 4e), and also peak force (Figure 4g), when loading is switched from one side of the structure to the other.

We use an auxiliary model following the same design principles as the asymmetric system to explain the cause of the locomotion in Figure 4c. Namely, one side is taller than the other, as caused by the attracting magnets after the first loading cycle. Furthermore, the less tall (left‐hand) side has Poisson's ratio close to zero, while the taller right‐hand side is strongly auxetic. The nonreversibility caused on the right‐hand side, by misalignment of the repelling magnetics during unloading (as in Figure 3b), is approximated as viscoelastic effects, while the left‐hand side is marginally viscoelastic. The auxiliary model global translations were 0.17 mm for Orientation 1 and 0.11 mm for Orientation 2, following the 2.4 mm applied compression over a single cycle. By multiplying these translations by 16.2/2.4 (i.e., deformation applied to structure/deformation applied to auxiliary model) and then by 20 (number of cycles), we can compare auxiliary model locomotion to the magneto‐mechanical structure considered in this work. As a result, Orientation 1 translation scales to 2.3 cm and Orientation 2 translation scales to to 1.5 cm, while the respective values for the structure were 2.4 and 1.3 cm ‐ Figure 4.

During the loading cycle, when the right‐hand side contracts (due to negative Poisson's ratio), the left‐hand side is free to slip (to the right). Recalling the geometry of the structure, where the right‐side is shaped like a wedge, applying compression causes a torque/rotation, that reduces reaction force underneath the left‐side of the structure ‐ allowing the left‐side to slip. At this point, frictional forces (and the relatively high reaction force on the right‐hand side) restrict (leftward) movement on the right of the sample. The line of transition between rightward and leftward deformation, caused by the transverse contraction of the right hand side of the structure, is near the center of this right‐hand‐side section. During unloading, hysteretic effects reduce the forces on the right‐hand side, and so also the torque (resulting from the wedged‐shape) ‐ meaning the left side of the structure is released to move downward. As such, the frictional forces under the left of the structure increase, and (leftward) recovery of the left‐hand side is smaller than during loading, while the (rightward) recovery of the right‐hand side is larger. When the direction of loading is switched from the top of the sample to the bottom (i.e., Orientation 1 to Orientation 2), the effects relating to movement of the plate, and recovery when the torque is released, act in opposite directions ‐ reducing the rightward shift.

The combination of the bias in geometry and effective mechanical properties, and the magnetically amplified nonreversibility that results in loss between loading and unloading, causes a large rightward shift during loading, and a smaller recovery to the left ‐ resulting in global translation. The locomotion of the structure can still be observed for a purely nonmagnetic structure given its biased structural design. For a detailed analysis of the locomotion related to the auxiliary nonmagnetic model the reader is referred to Section S5 (Supporting Information). In addition, in Section S6 (Supporting Information) it is shown experimentally that the locomotion can be also observed for the nonmagnetic version of the specific model considered in this work as long as one side of the structure would be locked while the other one would be free to deform.

Results presented in this work demonstrate great practical potential for the proposed concept to be applied in various applications that impact our lives on a daily basis. One potential direction is protective materials and sports applications, such as running shoes and helmets, that rely on efficient energy absorption.^[^ ^54^ ^]^ This stems from the fact that, as mentioned in the Introduction, the ability to control hysteresis/reversibility allows a transition between a low‐loss impact and a low‐impulse impact. Thus, the proposed concept makes it possible to construct versatile protective structures capable of changing their response mid‐performance. Similar tunability triggered by the nonreciprocal deformation process has been observed in terms of the structure's Poisson's ratio. As a consequence of a simple action, such as compression, the system can change its Poisson's ratio from being strongly negative to marginally positive. This ability could prove useful, for example, when attempting to customize already deployed biomedical devices, such as stents.

Perhaps the most promising application relates to soft robotics. The ability to construct miniaturized soft structures capable of exhibiting considerable locomotion, without being equipped in any elaborate engine‐like actuation mechanism, is a substantial challenge. There have been several attempts to deal with this problem; for example, via actuation by light^[^ ^45^ ^]^ or an external magnetic field.^[^ ^36^ ^]^ While both of these approaches are promising, they also have limitations. In the case of light actuation, constant access to a light source is required. Conversely, restricted access to light is a common reason to use soft‐robotics (i.e., in refined or remote conditions not suitable for humans). On the other hand, an external magnetic field typically has only one orientation, which makes achieving a biased internal actuation challenging. Here, we present solutions to these problems, by designing a system where the magnetic field can be internally controlled, and changes can be triggered by relatively weak compression. Thus, amongst many other possible examples, our mechanism could exhibit locomotion, for example, to move within tube‐shaped regions that exhibit cyclic compression / decompression cycles. One such example is blood vessels in the human body. However, in this case, further studies would have to be conducted to ensure the feasibility of the concept and reducing the risk of blocking the blood flow.

## 4 Conclusion

In this work, we proposed a magneto‐mechanical structure capable of not only following a controllable deformation process, but also breaking reversibility and reciprocity concurrently. We used these unique abilities to propose a system that can undergo a transition in its static mechanical properties, such as Poisson's ratio, from strongly negative to marginally positive. Similarly, the nonreversible deformation process was used in order to control hysteresis, linked to the energy absorption efficiency of the system. Finally, we induce a biased response in the system, leveraging these changes to Poisson's ratio and hysteresis to allow locomotion in response to cyclic loading/unloading, where dynamic nonreciprocity is also seen. These results demonstrate the potential of the proposed concept to be applied in many fields requiring adaptability, locomotive efficiency, and variable energy absorption efficiency, with prime examples being soft robotics and protective devices.

## 5 Experimental Section

### Experiments

The experimental prototype used in this work consists primarily of an elastic structure fabricated using a Formlabs 3 3D printer through the use of the Elastic 50A resin offered by the same company (*E* = 2.44 MPa, ν = 0.49, ρ = 1,010 *kg*/*m* ^3^ ‐ see Supporting Information S1). The sample had a sufficient out‐of‐plane thickness (*d* = 4 cm) to ensure that its deformation can be described from the perspective of a single plane. To avoid printing multiple prototypes for all of the considered types of arrangements of magnets, this structure was designed so that it was possible to enter removable yet tightly‐fitting cores fabricated from PLA filament into the square‐like structural blocks (see Supporting Information). These cores had thin cylindrical N38 neodymium magnets permanently glued to their surface. The magnets had a diameter *d*_*m*_ = 1 cm, height *l*_*m*_ = 1 mm, and residual induction of ∼1200 mT. All of the magnets had the same dimensions described in the Concept section.

To analyze the static mechanical properties, the structure was subject to two loading cycles through the use of the Shimadzu EZ‐SX Short testing machine equipped with a 500N load cell. The maximum applied displacement‐controlled deformation during each of these cycles was set to 14 mm while a constant deformation rate of 20 mm/min was set, which (due to the viscoelasticity of the polymer) was artificially lowered from various rubber test standards such as ASTMD638‐14 and BSEN ISO 3386‐1,^[^ ^60^, ^61^ ^]^ and equated to an effective engineering strain rate of 0.005 s^−1^. On the other hand, to analyze the locomotion of the structure with the biased design, 20 dynamic loading/unloading cycles were applied. Here, the maximum vertical deformation was set to be 16.2 mm, while the constant deformation rate was equal to 15 mm/s.

### Simulations

Numerical simulations (in ANSYS Mechanical) generally matched the quasistatic experiments. The model was simulated as a thin (0.2 mm) sheet, with out‐of plan motion constrained on one side, facilitating efficient simulations with stable contacts.^[^ ^62^ ^]^ These contacts were represented as frictionless within the body of the metamaterial‐based model, while a coefficient of friction of 0.3 was applied externally. An isotropic linear elastic material model approximated the properties of the resin in the experiment. Hexahedral solid elements with a minimum size of 0.5 mm were applied, with refinements of 0.2 mm across thin walls and close to contacting surfaces, reduced to 0.1 mm for the hinge between the inner, bottom rotating square, and the oblique rib. These were selected based on a mesh sensitivity study between applied force and number of elements. Quasistatic deformations of 12.75 mm (reduced to 12 mm for the repelling magnets) were applied to rigid plates placed above the sample, while symmetry was set through the sample center, and along the bottom surface. Large deformations were enabled, with a minimum time step of 0.001 s. A semi‐implicit solver was used, such that before self‐contact between structural elements, all simulations were solved implicitly. This was then switched to an explicit method if local dynamics relating to stick/slip between such elements caused rate‐dependent motion and unstable solutions.

The magnets were represented as time‐varying forces applied to surfaces, based on analytical calculations of magnet coordinates and orientations. The force between magnetic dipole moments representing two magnets (**m_1_** and **m_2_**) is:^[^ ^58^, ^59^ ^]^

(1) $$\begin{array}{cc} & f_{l_{\left(r,m_{1},m_{2}\right)}}\\ & \;=\frac{3\mu_{0}}{4\pi r^{5}}\left[\left(m_{1}\cdot r\right)m_{2}+\left(m_{2}\cdot r\right)m_{1}+\left(m_{1}\cdot m_{2}\right)r-\frac{5\left(m_{1}\cdot r\right)\left(m_{2}\cdot r\right)}{r^{2}}r\right] \end{array}$$

whereby the resultant distance *r* = |**r**|, with *r*_*x*_ = 0, *r*_*y*_ = *sin*θ(*lcos*θ + 2*t*), and *r*_*z*_ = *lsin* ^2^θ + 2*tcos*θ, θ is the angle between a rotating square and the y‐axis, *l* is the distance from the magnet center to the hinge, and t is the depth of the center of the magnetic pole (0.33 mm) plus the minimum separation (of 0.5 mm, when held apart by repelling magnets). The electromagnetic permeability of air μ_0_ is 1.257 × 10^−6^ *H*/*m*, while scalar scalar x, y, and z components of both magnetic dipoles are *m*_1_ & *m*_2_ = 5 × 10^−4^ *Am* ^2^. While the attracting magnets interact and then close, meaning point representation was sufficient, the repelling magnets did not. For these, **r** changed as *l* varies across the diameter of the magnets:

(2) $$f=A_{m}^{-1}\int C_{\left(l\right)}f_{l_{\left(r,m_{1},m_{1}\right)}}\;dl$$

where *C*_(*l*)_ is the chord (in the x‐axis) of the magnet at position *l*, and *A*_*m*_ is the total area of the magnet. The magnetic dipole was segmented during the integration (giving scalar x, y, and z components of $2\;r\;m_{1\&2}$ = 5.5 × 10^−5^ *Am*). The y‐axis forces were < 5 % of the z‐axis, and so were entered as incremental values (every 20 s). A surrogate was fit between z‐axis force (F) and time (T) (for coefficients, see Supporting Information S3), using two‐term Gaussian functions (giving NRMSE < 1 %):

(3) $$F_{T}=\sum_{n=1}^{n=2}a_{n}e^{-{\left(\frac{T-b_{n}}{c_{n}}\right)}^{2}}$$

The temporal magnetic force was artificially increased (by a factor of 1.5) for the magnet pairing closest to the sample center (as a total of three magnets were used here, rather than two). As the outer (repelling) magnets did not touch during the simulations (Figure 3b), their force was reduced by a factor of 1.67. An analytical model was also developed, to define a direct relationship between magnetic interactions (Equation 1) and the underlying structure. This is described and compared with experimental data in Section S4 (Supporting Information). A Matlab implementation of this analytical model is also included as a supplementary file.

A simplified/auxiliary numerical model (in ANSYS Mechanical) was used to explain the walking mechanism. This simulation used similar settings to the previously described ones and a geometry that represented the shape of the sample after the first loading cycle (see Supporting Information S2). Separate elastic and viscoelastic material models were respectively applied to the left and right side of the representative geometry, with the right hand side assigned a viscous contribution (consistent with Figure 3). This was a one‐term Prony series with a relative shear modulus of 0.5 after 1 s. The left‐hand side was given a diminished viscoelastic model ‐ a one‐term Prony series with was a relative shear modulus of 1 after 10 s. Poisson's ratio was set to zero on the left‐hand side, and –0.9 on the right (as in Figure 2). Young's modulus of both sides was arbitrarily set to 1 MPa. Stiff plates compressed the sample by 2.4 mm (for a 0.2 s load/unload cycles), with a static and dynamic frictional co‐efficient of 0.2 applied to the bottom surface, and 0.05 to the top surface (reflecting the smaller contacts in the model than the representative geometry).

## Conflict of Interest

The authors declare no conflict of interest.

## Author Contributions

K. K. D. led the conceptualization, experiments, data analysis, and writing. O. D. contributed to the conceptualization, supported the experiments, performed data analysis and simulations, and assisted with writing. J. A. I. M. was involved in conceptualization, as well as reviewing and editing. M. K. contributed to conceptualization, reviewing, and editing.

## Supporting information

Supporting Information

Supplemental Video S1

Supplemental Video S2

Supplemental Video S3

## Data Availability

The data that support the findings of this study are available from the corresponding author upon reasonable request.
