# Supplementary material for: Mechanical Metamaterial‐Based Structure with Magnetically Controlled Nonreversibility and Nonreciprocity for Programmable Locomotion
Source: Adv Sci (Weinh). 2025 Jun 19;12(32):e03088. doi: 10.1002/advs.202503088 (PMC12407254; doi:10.1002/advs.202503088)
Supplement: Supplementary file 1 — Supporting Information [file ADVS-12-e03088-s002.pdf]

ADVANCED SCIENCE
Open Access

Supporting Information

for *Adv. Sci.*, DOI 10.1002/advs.202503088

Mechanical Metamaterial-Based Structure with Magnetically Controlled Nonreversibility and Nonreciprocity for Programmable Locomotion

Krzysztof K. Dudek, Olly Duncan*, Julio A. Iglesias Martínez and Muamer Kadic**

Supplementary Materials for: Mechanical Metamaterial-based Structure with Magnetically Controlled Nonreversibility and Nonreciprocity for Programmable Locomotion

Krzysztof K. Dudek [1,2,*,†], Olly Duncan [3,*,†], Julio A. Iglesias Martínez [4], Muamer Kadic [2,*]

1)Institute of Physics, University of Zielona Gora, ul. Szafrana 4a, Zielona Gora, 65-069 Poland
2)Université Marie et Louis Pasteur, Institut FEMTO-ST, 25000 Besançon, France
3)Manchester Metropolitan University, Department of Engineering, Manchester, United Kingdom
4)Institut Jean Lamour, CNRS UMR 7198, University Lorraine, 54011 Nancy Cedex, France
*e-mail: k.dudek@if.uz.zgora.pl, O.Duncan@mmu.ac.uk, muamer.kadic@femto-st.fr
†These authors contributed equally.

S1) Formlabs Elastic 50A Resin Material Model

A hyperealstic material model was characterised, to allow appropriate selection for the finite element model. Standard test samples were made to characterise Formlabs elastic 50A resin [1] for stereolithography apparatus (three of tensile type-iv ASTMD638-14 - [2], and three of 20 mm diameter 10 mm thick disks for BSEN ISO 3386-1 compression tests [3]). These were made in a Formlabs Form 3.

Sample dimensions (Vernier Calipers) and masses (Sartorius, AC210S) were recorded, and were similar to the expected density (1010 kg/m^3 [1]). Quasistatic tensile tests were undertaken to 100% tensile (engineering) strain on a mechanical test device (Tinius Olsen 50ks, 1 kN load cell, sampling at 15 Hz) at a strain rate of 0.005 s^{-1}, after applying a 0.5 N pre-load. Compression tests were undertaken on the same device, up to the same strain and strain rate. Speckle patterns for digital image correlation (DIC) were applied using a black permanent marker before the tests, then tests were filmed with a DSLR camera (Nikon D3200, 1200 × 1080p, 24 Hz).

Stress was obtained from force data from the mechanical test device and sample dimensions, while full-field logarithmic strains were obtained by 2D DIC (GOM Correlate 2019). These were aligned by visually identifying the start of test, aligning time signatures, and interpolating deformations using a linear relationship between time and axial displacement. Young's moduli were obtained as the gradient of straight lines fitted up to 5% strain of obtained stress vs. strain data (Figure S1a). Stresses (e.g., $\approx 1MPa$ at 50 % elongation) were similar to those in the materials data sheet [1]. Poisson's ratio was measured as the negative of the gradient of a line fitted to axial vs. transverse strain (up to 10% tensile strain - Figure S1b).

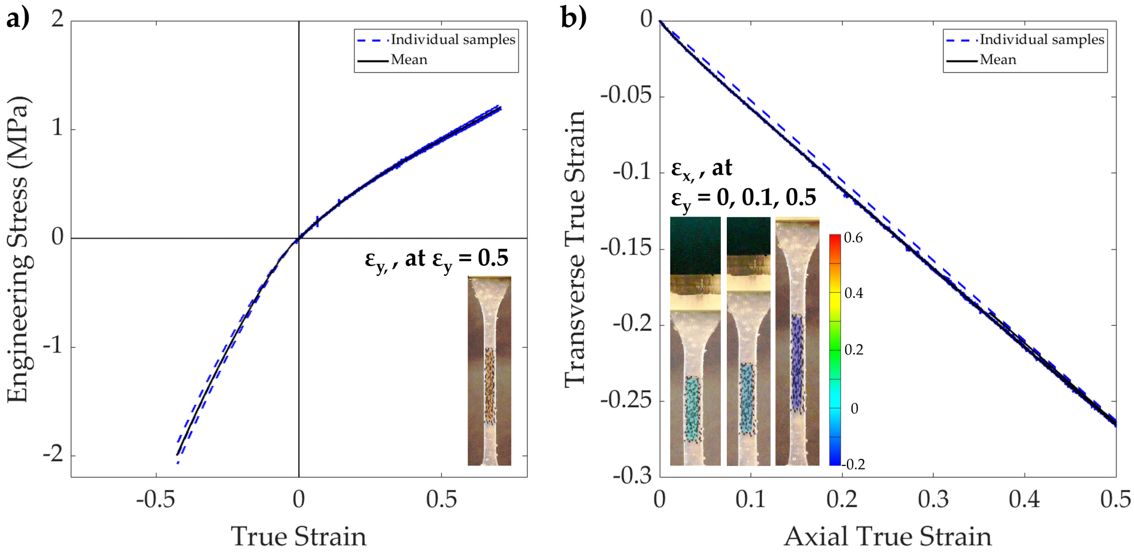

Figure S1: *a) stress vs. strain data, b) axial vs. transverse strain data. Inserts in show DIC contour plots of a) axial and b) transverse strain (same legend for both).*

Mean engineering stress vs. true strain data was used to fit a three term Mooney Rivlin hyperelastic incompressible material model [4] (which was found to match the data, Figure S2a) .

$$W = \sum_{i=j=0}^{n} C_{ij}(I_1 - 3)^i (I_2 - 3)^j \tag{S1}$$

where the material was assumed incompressible, W is the strain energy, i and j are constants defining the number of terms (between 0 and n = 1), with $i + j \leq 2$), C_{ij} are co-coefficients $C_{00} = 0$, and I_1 and I_2 are the Cauchy strain invariants:

$$I_1 = \lambda_1^2 + \lambda_2^2 + \lambda_3^2 \tag{S2}$$

$$I_2 = (\lambda_1\lambda_2)^2 + (\lambda_2\lambda_3)^2 + (\lambda_1\lambda_3)^2 \tag{S3}$$

where $\lambda_{1,2,3}$ are stretches ($\epsilon + 1$). The Cauchy stress is [4]:

$$\sigma_1 = 2\left(\lambda_1^2 - \frac{1}{\lambda_1^2\lambda_2^2}\right)\left(\frac{\delta W}{\delta I_1} + \lambda_1^2 \frac{\delta W}{\delta I_2}\right) \tag{S4}$$

The engineering stress σ_1^e is the product of σ_1 and λ_2^2.

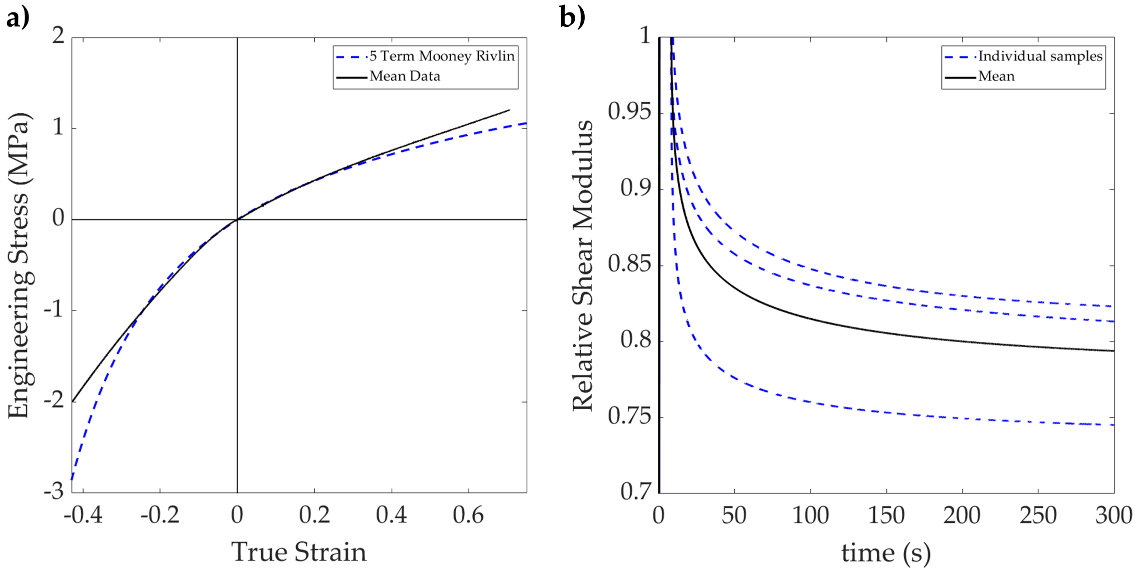

Figure S2: *a) Hyperelastic material model, and data used to fit it b) Prony series data (normalised to obtained shear modulus of 1.40 MPa)*

Load/hold tests were also undertaken up to 20% compression with a loading strain rate of 5 s^{-1} to fit a four term Prony series (Figure S2b), to approximate the viscoelastic response of the TPU [5]:

$$G_t = G_0\left(\alpha_\infty + \sum_{i=1}^{n=4} \alpha_i e^{-\frac{t}{\beta_i}}\right) \tag{S5}$$

where α is the relative shear modulus, the respective 0 and ∞ subscripts denote t = 0 and t = ∞ relative moduli, β was relaxation time, t was time, and i denotes the number of terms (i.e., 4). Five cycles of compression were undertaken immediately before the tests, to mitigate the Mullins effect. Relative shear modulus was obtained from the defined strain, stress, and previously measured Poisson's ratio:

$$G = \frac{E}{2(1+\nu)} \tag{S6}$$

The disks were then impacted with a flat plate on a bespoke, guided mass drop rig [6], at 1 J (m = 2.68 kg, v = 0.86 m/s), causing high strain rate compression. To check the material model, these impacts were simulated (LS-Dyna, via. Ansys Workbench 2023 R1), by applying 1 mm of compression (using rigid plates), to a disk of the hyperelastic material. Hexahedral solid elements were used, with minimum element size set to 1 mm. For both the rigid body contacts (between plates and honeycomb), the segment-based contact setting, soft = 2 was applied [5]. Frictional contacts $\mu = 0.7$ were defined, with a damping

Table S1: Material properties and models

Material Model	Co-efficient Value		
**Mooney Rivlin (3-term) (MPa)**			
C_{10}	0.409		
C_{01}	0.040		
C_{11}	-0.013		
**Prony Series**	$\alpha_i(MPa)$	β_i	
i=1	0.039	64.8	
i=2	0.071	4.41	
i=3	0.043	0.092	
i=4	0.038	0.027	
**Properties**			
$\rho(kg/m^3)$	$E(MPa)$	ν	$G(MPa)$
1010	2.44	0.5	0.819

stabilisation factor of 0.1. A time-step safety factor of 0.5 was used, with a maximum number of cycles of 10^6, over the 0.6 ms simulations. The default hourglass control (Flanagan–Belytschko Stiffness Form with exact volume integration; LS-DYNA ID (5) of 0.10) was used, with the default settings of quadratic bulk (1.5) and linear bulk (0.06). Close fit was seen between the numerical model and the test data (Figure S3). The material properties and models are shown in (Table S1).

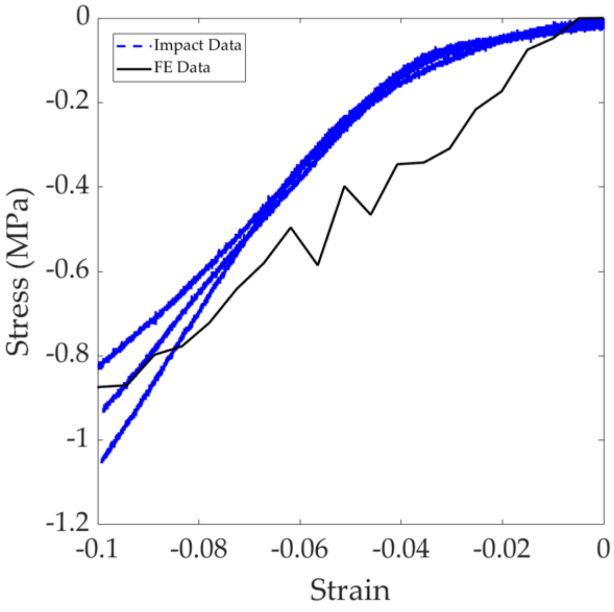

Figure S3: *Stress vs. strain from impacts and simulations of equivalent rate compression tests.*

S2) Sample Geometries

The geometries of the metamaterial, and the approximated outer dimensions when attracting magnets fix the rotating squares are shown in (Figure S4). The solid section on the inserts were the same width as the magnets (10 mm).

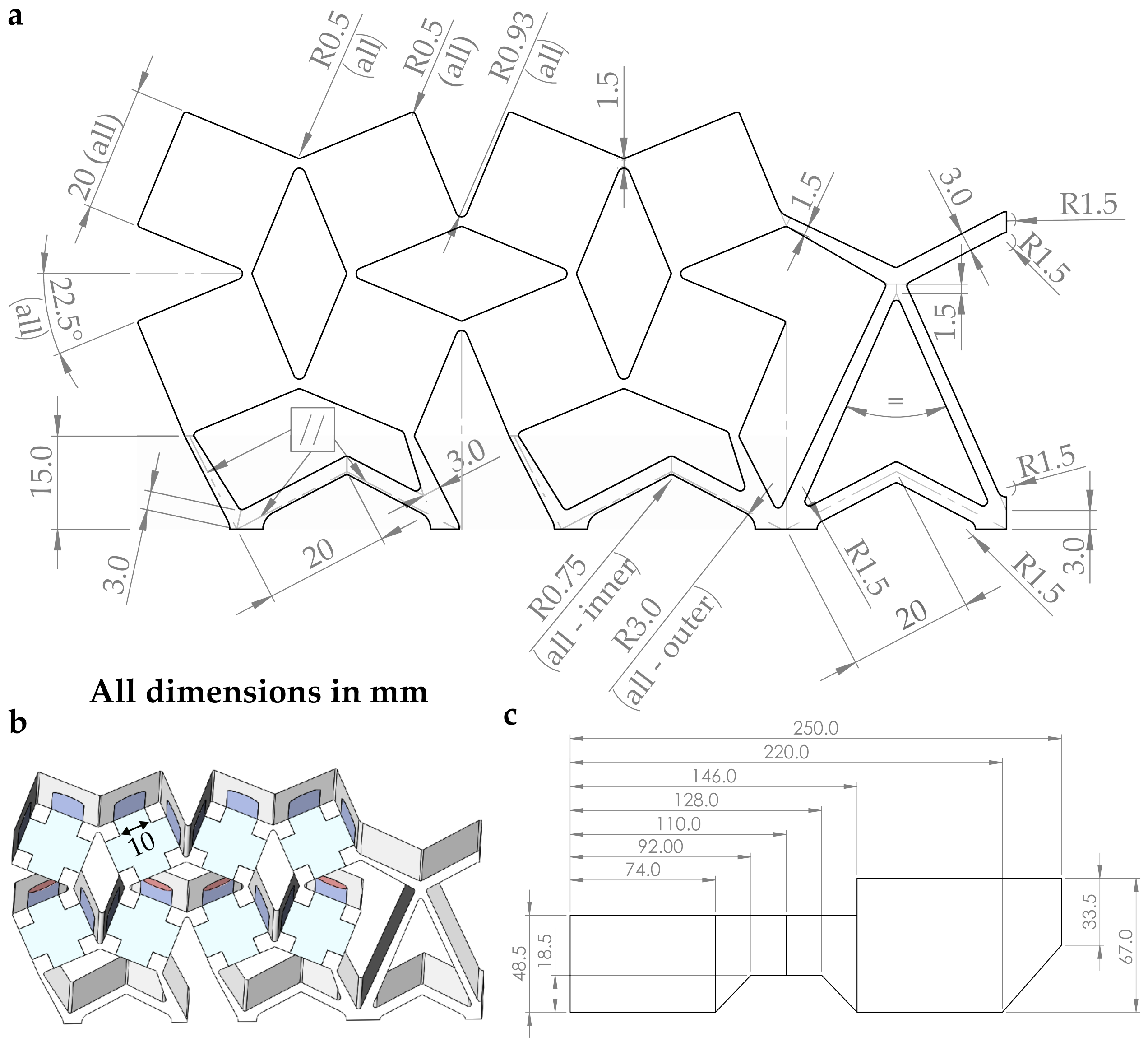

Figure S4: *Engineering drawings of a) the metamaterial-based structure, with the shading showing rotating squares, the laterally connecting ribs, and the vertically connecting ribs, noting that the depth was 40 mm, height was 67 mm, and width was 140 mm, b) the location of removable inserts (light blue) and magnets (light red), and c) the approximated geometry used in the auxiliary model to explain the walking mechanism.*

S3) Magnetic forces

The calculated values for magnetic forces, and the result of the two-term Gaussian curve fits, are shown in Figure S5. The coefficients for the Gaussian curve fits are shown in Table S2, relating to equation 3 in the manuscript.

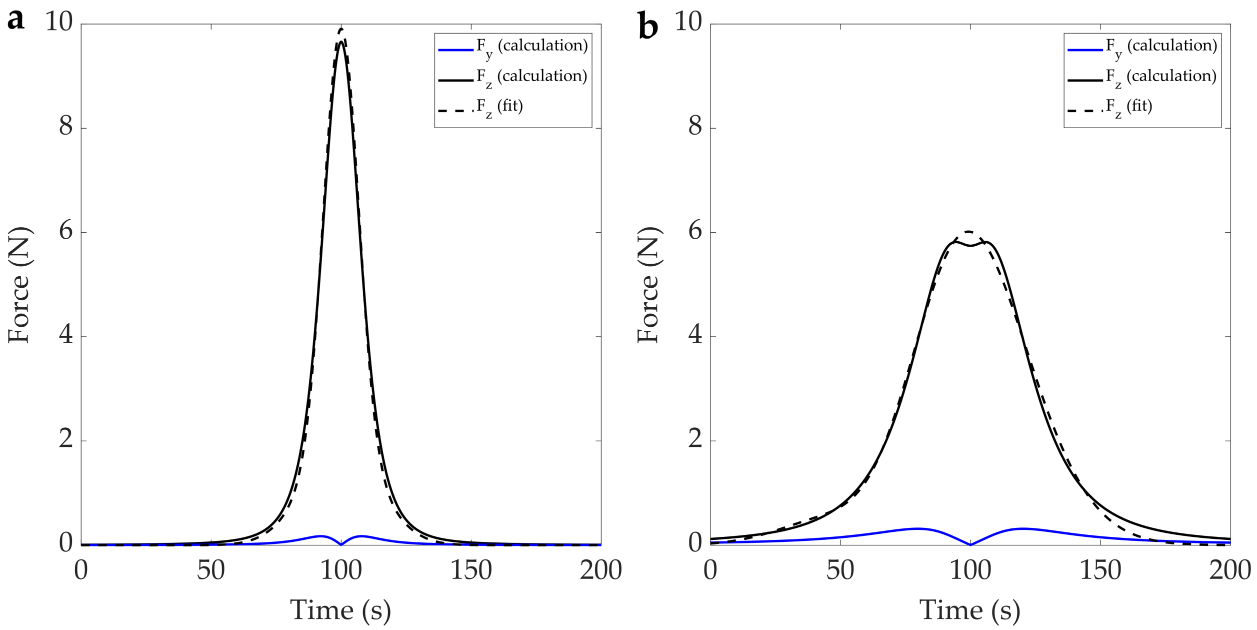

Figure S5: *The calculated and fit magnetic forces vs. time for a) attracting and b) repelling magnets.*

Table S2: Coefficients for magnetic force response

	**Co-efficient (n = 1)**	**Co-efficient (n = 2)**
**Attracting**		
a	0.020	0.005
b	100	100
c	9.38	20.2
**Repelling**		
a	0.020	-0.009
b	89.2	71.2
c	38.6	27.3

S4) Generalised analytical model

The responses seen in the proposed structure are caused by interactions between three components (Figure S6a): Firstly, the magnets change the effective properties of the rotating squares by modifying the force constants associated with hinging between squares. Then the effective stiffness and Poisson's ratio differences between the section with rotating squares and the surrounding structure lead to subsequent changes in effective properties under large deformations, or when the attracting magnets lock the rotating squares. To assist with future design of systems like this, we apply these interactions in an analytical model.

Firstly, the rotating squares section is modelled kinematically according to established methods [7]. The deformation of the rotating squares is dominated by hinging between the squares, whereas the squares themselves are assumed to be rigid. Using the parameters / dimensions introduced in Section S2 (Figure S4a), the dimensions X_{1r} & X_{2r} of the rotating squares can be obtained as a function of side length $a = 20mm$ and orientation θ (whereby the angle between adjacent squares is defined as 2θ), as well as the number of squares in each axis:

$$X_{1r} = 2X_{2r} = 4a(cos\theta + sin\theta) \tag{S7}$$

As the elastic medium is stiffer than the rotating squares, this causes the following changes to outer dimensions X_1 & X_2:

$$X_1 = X_{1r} + X_{1e} \tag{S8}$$

$$X_2 = X_{2r} + X_{2e} \tag{S9}$$

whereby X_{1e} & X_{2e} are the dimensions of the elastic medium. We now define a vector with values of θ_i that reduce from $\theta_0 = 22.5°$ to 0. Logarithmic strains ϵ_x & ϵ_y are:

$$\epsilon_{\mathbf{x}} = \mathbf{ln}(\mathbf{X_{1(i)}}/\mathbf{X_{1(0)}}) \tag{S10}$$

$$\epsilon_y = ln(X_{2(i)}/X_{2(0)}) \tag{S11}$$

Incremental Poisson's ratio is:

$$\nu_{yx(i)} = -\frac{\delta\epsilon_{x(i)}}{\delta\epsilon_{y(i)}} \tag{S12}$$

Force constants for hinging between squares k_h can be defined by approximating the hinges as bending beams with second moment area $I = dt_h^3/12$:

$$k_{h(i)} = E_s I/(lcos\theta(i)) \tag{S13}$$

whereby E_s is the modulus of the intrinsic polymer. Two types of hinge appear in the structure - those between the cubes have a mean thickness t_h of 2 mm, and those between the squares and the elastic surround have a mean thickness of 2.5 mm. The sample depth (d) is 400 mm. The two respective hinging constant are $k_{h(1)}$ & $k_{h(2)}$.

The force produced by each magnet f_m, and the relative distance between them, is defined in the same way as in the manuscript (Equation 1). To define a force constant for this interaction, the vertical force produced by magnetic interaction $f_{m,y}$ is divided by displacement increments k_m:

$$k_{m(i)} = f_{m(i),y(i)}/(\Delta X_{2(i)}) \tag{S14}$$

Now, counting the number of internal hinges $N_1 = 10$, external hinges $N_2 = 5$, and magnets $N_3 = 8$, a function for tangent modulus can be defined:

$$E_{(i)} = (N_1 k_{h(1)(i)} + N_2 k_{h(2)(i)} + N_3 k_{m(i)})/A \tag{S15}$$

whereby $k_{m(i)}$ is negative when the magnets are attracting, and A is sample cross sectional area $X_{1(i)} \times d$. This is combined with the elastic substrate after the rotating squares reach the point of self-contact, which

has undeformed tangent modulus (E_y) of 15 kPa, increasing to 50 kPa when it is compressed to a local strain of 50 % (outer strain of 10 %).

At the increment where the tangent modulus becomes negative (for the case of the attracting magnets), a jump to the state of magnets being closed ($\theta = 0$) occurs. Here, the incremental Poisson's ratio is therefore extremely high in magnitude - although to reflect the fact that each set of magnets closes individually and not all at the same time, this value is divided by four.

To construct a comparative test-case to that in the manuscript (Figures 3 and 4), a moving mean is applied to tangent modulus and Poisson's ratio data, over 4% strain increments, reflecting the increments measured in the experiments. Noting that while frictional and rate dependent effects, that restrict Poisson's ratio are disregarded, as is the slow closing of the magnets, the same trends as seen in the manuscript are observed (Figure S6b & c). The reader is referred to the Matlab implementation of this analytical model, that is included as in the Supplementary Materials.

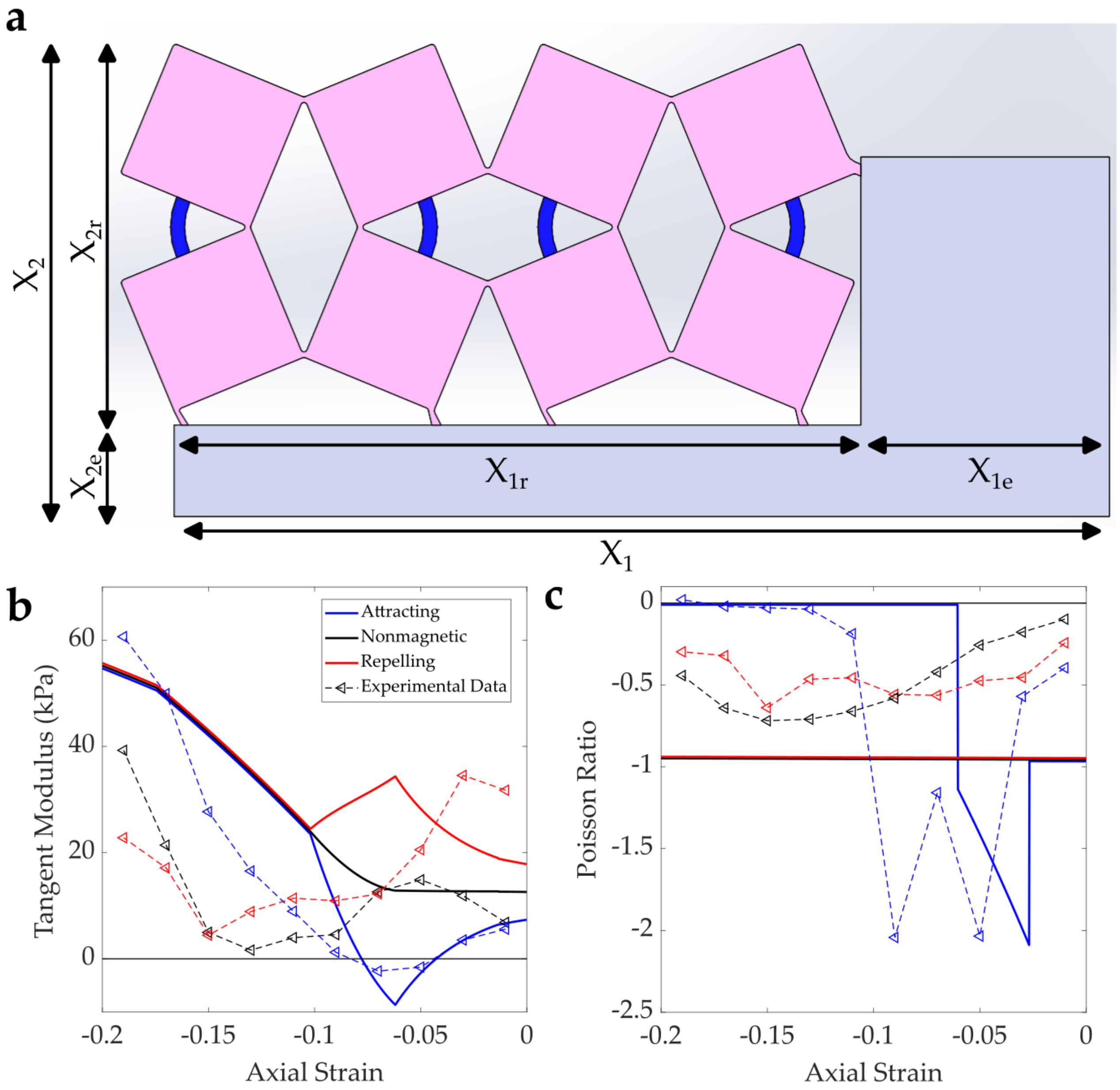

Figure S6: *a) Schematic showing the segmented structure consisting of rotating squares (pink), magnets represented as effective springs that modify force constants around hinges (blue), and the elastic medium these rotating squares are embedded in (grey/blue), b) Tangent moduli vs. axial (true) strain, with comparison to experimental data, and c) Poisson's ratio vs. axial strain (same legend for b) and c)).*

S5) Analysis of locomotion - simulations

So that the locomotive concept can be generalised for design purposes, we first analyse the contribution of effective properties. To do this, we use the auxiliary model presented in the manuscript (in Figure 4). Rather than specifying different effective properties in different zones, we applied homogeneous properties that span the range of those in the manuscript. To allow recovery, we obtain x-axis deformation in the centre of the sample 10 seconds after the applied load/unload cycle. For all cases, a positive Poisson's ratio caused leftward locomotion, a negative Poisson's ratio caused rightward locomotion, and a Poisson's ratio of zero caused little to no locomotion (Figure S7).

Viscoelasticity (Figure S7b) generally led to more locomotion than the elastic example (Figure S7a), or the partially viscoelastic example (Figure S7c, other than for the zero Poisson's ratio case, where rightward locomotion was seen). The magnitude of locomotion was also always greater for Orientation 1 (when the top plate was moving) than for Orientation 2. At a small deformation ($t = 0.01$ s), Figures S8a & b show that the left hand side moves toward the right, and the right hand side moves leftward. The transition between left and right deformation is close to the centre of the right hand section - which is held in place by the compression plates. The direction of deformation is then reverse during the unloading phase ($t > 0.1$ s). Toward the end of the unloading phase, the line marking the transition between positive and negative x-axis velocity moves toward the left of the sample (Figure S8b) - i.e. the whole sample moves to the right.

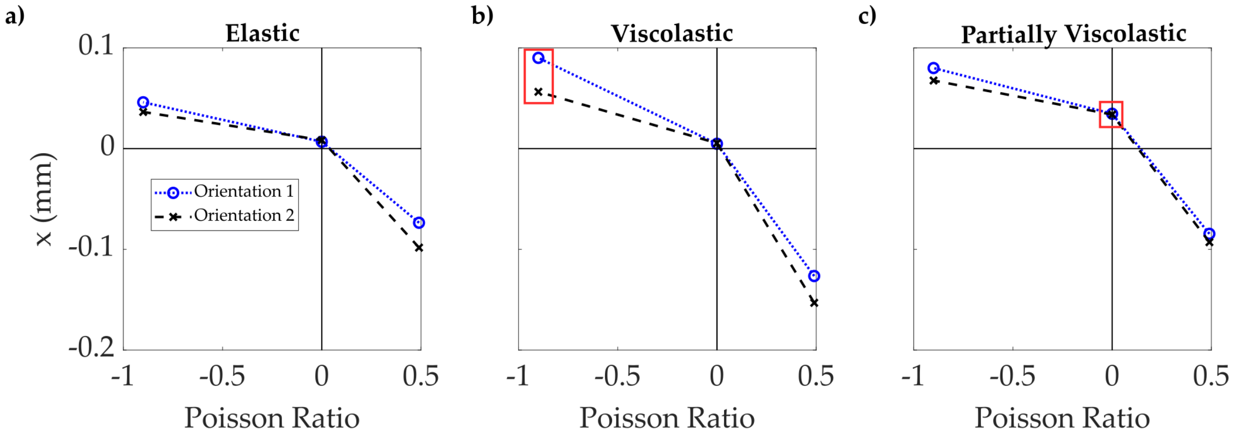

Figure S7: *Simulation outputs using the auxiliary model, with a) elastic properties specified, b) a Prony series included (with the same inputs as those used for the RHS in the manuscript), and c) a Prony series included (with the same inputs as those used for the LHS in the manuscript). While Poisson's ratios are varied, Young's moduli are all set to 1 MPa. Orientation 1 and Orientation 2 relate to whether the compressive deformation is set on the top plate or the bottom plate. The red boxes highlight where effective properties match those used in the manuscript (RHS is shown in b) and LHS in shown in c)).*

To explain the causes of the locomotion, we study Orientation 1, while matching the the effective properties used in the manuscript, which also cause the most rightward locomotion for zero and negative Poisson's ratios (see red boxes in Figure S7b & c).

Observing the contact pressure on the left hand side of the sample (Figure S8c), this generally centres around 1.1 kPa, which is caused by gravity acting on the sample. As the sample is compressed in Orientation 1, this value reduces. Considering the wedged shape on the right side of the sample, the larger top surface will cause a torque (annotated in Figure S8a). This torque causes a clock-wise rotation of the sample, reducing the pressure between the left of the sample, and the bottom plate. The viscoelasticity of the constituent materials, and other loss factors such as friction, lower the contact pressure on the right of the sample during the unloading phase (Figure S8b). As such, in the case of the auxiliary model, first-order time dependence causes the left of the sample to show a higher reaction force/pressure during the unloading phase, meaning that traction (due to friction) would also increase, pushing the sample to the right. In the considered structure, this nonreversibility is not only related to the rate dependence. It is also related to minor changes in relative magnetic orientation between the load an unload phase (See Figures 2 and 3 in the manuscript).

The torque only partially explains the change in pressure on the left of the sample (and so the locomotion). During the unloading phase, the pressure on the left of the sample is greater than that caused by gravity . Quasistatically, this would not be expected to switch direction entirely between the load and unload phase.

This is due to the energy stored in the sample, due to this torque, being released - causing downward acceleration of the right hand side (Figure S8e).

The observed nonreciprocity, i.e. change in translation when the applied deformation is switched from the top plate to the bottom, is also related to such second order time dependence. The left-side contact pressure for Orientation 2 is similar to that of Orientation 1 - although it is slightly positive at the start of the test (when the plate is causing upward acceleration of the whole sample), and lower in the unloading phase (when the downward acceleration of the sample reduced the resultant force). These smaller differences in left-side contact pressure between the load and unload phase then also explain the reduction in observed locomotion.

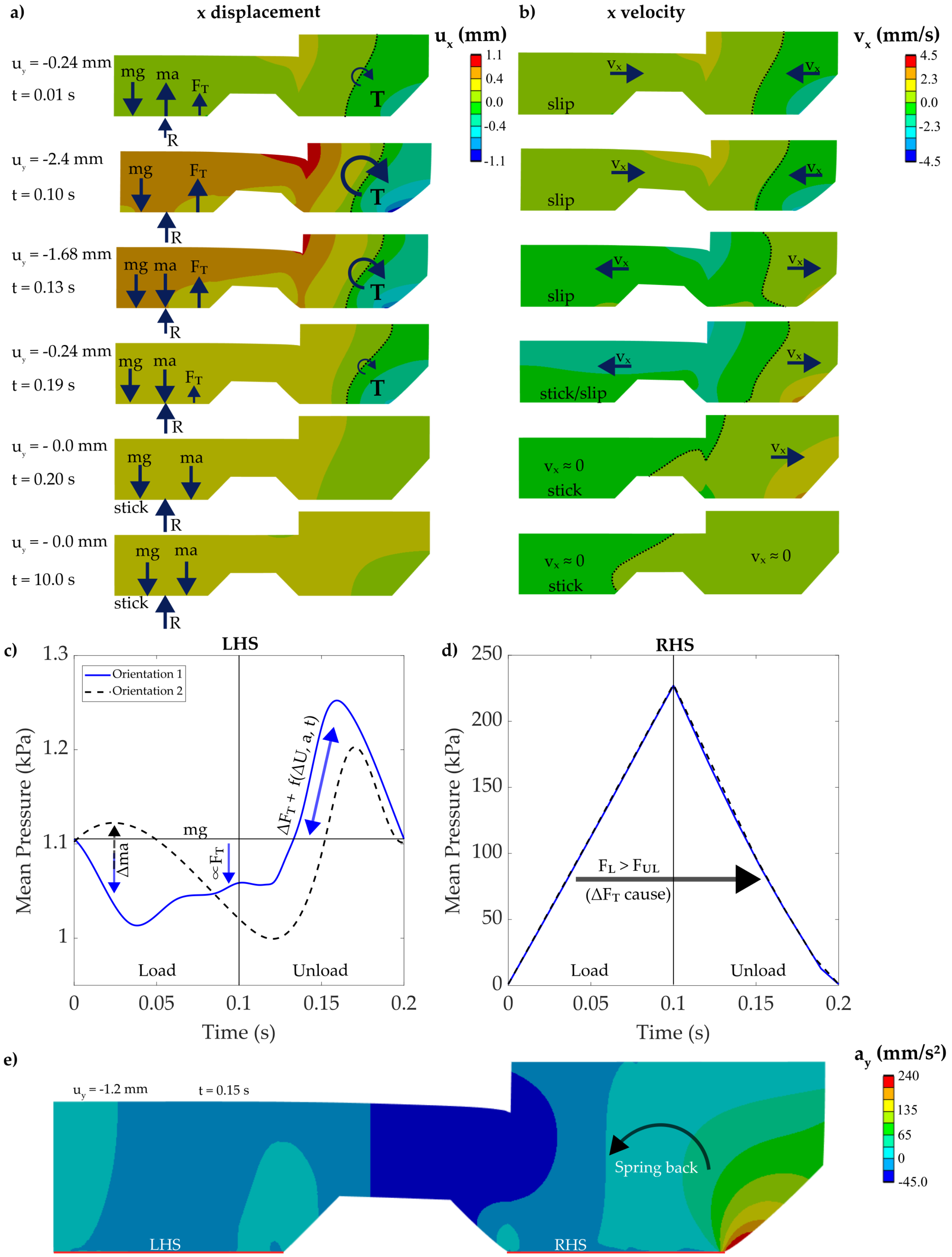

Figure S8: *Simulation outputs using the auxiliary model. a) & b) show contour plots of a) x-deformation and b) x- velocity, at time increments shown in a), each using separate scale bars (top right). These are segmented to show the transition between positive and negative deformation/velocity, while annotations are added to a) to show the causes of resultant forces on the left-sides bottom surface, and note where these cause the model to (mostly) stick to the bottom plate. c) and d) mean pressure on the c) left and d) right side (bottom surfaces). e) contour plot of y-axis acceleration at 0.15 s, with red lines and labels showing the location of contact pressures from c) and d). F_T is the LHS reaction force caused by the torque (T), R is reaction force, and F_L & F_{UL} are loading and unloading forces, respectively.*

S6) Analysis of locomotion - experiment

In the previous section, based on the auxiliary theoretical model and computer simulations, it has been explained that the effect of locomotion of the considered model originates from its nonuniform geometry that can be observed once one the rotating squares on one side of the structure are locked. Thus, to observe the nonzero translation, the system could in principle be nonmagnetic as long as one would employ a mechanism to lock the rotating squares on one side. To show that this can be achieved, and that this principle also applies to our specific model, we conducted an experiment using 10 compression cycles, where one side of the system is truly nonmagnetic while the other side is locked throughout all compression cycles due to magnetic attraction between the adjacent square-like elements. As shown in Fig. S9, even in this case, where magnetic forces are not affecting the deformation process beyond locking one group of structural elements, it is possible to observe a considerable extent of locomotion equal to 4.27 mm.

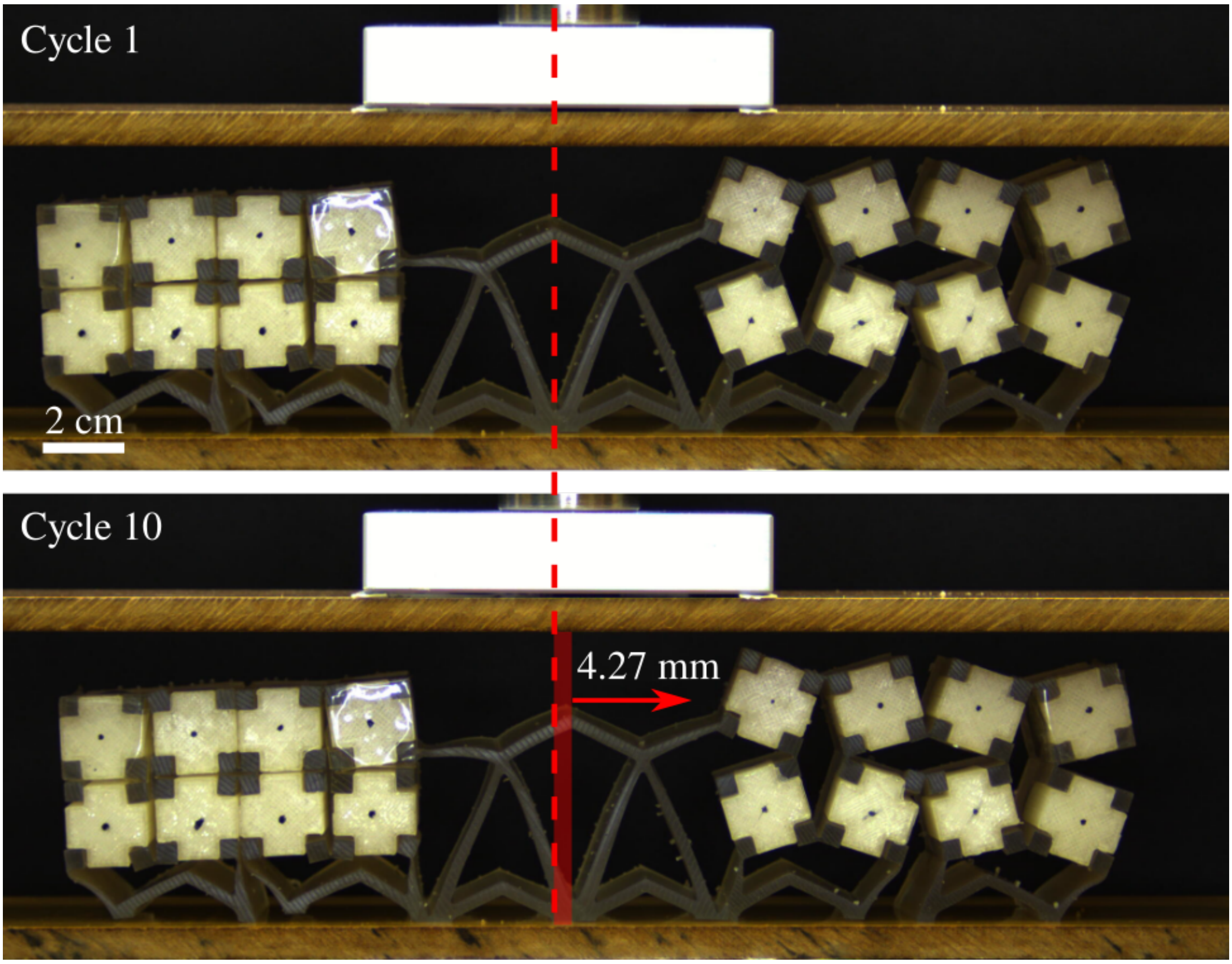

Figure S9: *Experimental analysis of the prototype locked on one side that was subjected to 10 compression cycles. The right-hand side of the structure is fully nonmagnetic.*

References

[1] Formlabs. *Elastic 50A Resin V2 Resin for Soft Flexible Parts Material Data*. 2024. URL: https://media.formlabs.com/m/4acd3cb149be1674/original/-ENUS-Elastic-50A-V2-TDS.pdf.

[2] American Society for Testing and Materials. "ASTM D638 - 14: Standard Test Method for Tensile Properties of Plastics". In: *ASTM International* 82.C (2016), pp. 1–15. ISSN: 21653992. DOI: 10.1520/D0638-14.1.

[3] *BS EN ISO 3386-1:1997+A1:2010: Polymeric materials, cellular flexible. Determination of stress-strain characteristics in compression Low-density materials*. 2010.

[4] R. S. Rivlin. "Large Elastic Deformations of Isotropic Materials". In: *Philosophical Transactions of the Royal Society of London. Series A, Mathematical and Physical Sciences* 240.822 (1948), pp. 459–490. DOI: 10.1007/978-1-4612-2416-7_8.

[5] Todd Shepherd et al. "Validation of a Finite Element Modelling Process for Auxetic Structures under Impact". In: *Physica Status Solidi B: Basic Solid State Physics* 1900197 (2020).

[6] M. F. Parisi et al. "Indentation and impact response of conventional, auxetic, and shear thickening gel infused auxetic closed cell foam". In: *Smart Materials and Structures* 32.074004 (2023). DOI: 10.1088/1361-665X/acd91c.

[7] J. N. Grima and K. E. Evans. "Auxetic behavior from rotating squares". In: *Journal of materials science letters* 19 (2000), pp. 1563–1565.
